# Nurse and Physician Stakeholder Guidance on Improving Sexually Transmitted Infection Care in a Pediatric Emergency Department

**DOI:** 10.5811/westjem.61866

**Published:** 2026-09-09

**Authors:** Roland C. Merchant, Daniela Ramirez-Castillo, Arlet Capeta Romero, Rachel Solnick, Patricia Mae Martinez, Christopher Strother, Melissa A. Clark

**Affiliations:** * University of South Florida, Morsani College of Medicine, Department of Emergency Medicine, Tampa, Florida; † Icahn School of Medicine at Mount Sinai, Department of Emergency Medicine, New York City, New York; ‡ Brown University School of Public Health, Department of Health Services, Policy and Practice, Providence, Rhode Island

## Abstract

**Introduction:**

Pediatric emergency departments (PEDs) and general EDs in the United States (U.S.) do not consistently deliver sexually transmitted infection (STI) care that aligns with clinical guidelines. To inform the development of an implementation strategy plan aimed to improve STI care in a PED, we interviewed PED nurse and physician stakeholders to identify challenges and corresponding potential solutions aimed to improve STI care in a PED.

**Methods:**

We conducted semi-structured interviews with 27 stakeholders consisting of emergency medicine (EM) residents, pediatrics residents, pediatric EM (PEM) fellows, attending pediatric emergency physicians, and PED nurses at an academic urban PED. Using the Tailored Implementation in Chronic Diseases (TICD) framework, we prepared semi-structured interview guides specific to stakeholder roles structured according to the usual six successive workflow stages of STI care in the PED (triage, history-taking, physical examination, testing, treatment, and follow-up). Questions concerned current practices on providing STI care, challenges encountered, and potential solutions. Interviews were transcribed and analyzed using rapid content analysis. Responses were coded independently by two reviewers and categorized by TICD determinant domain.

**Results:**

Participants identified multilevel implementation challenges across the stages of STI care in the PED. Structural challenges included lack of privacy and limited private exam rooms, inadequate STI-related workflows, and inadequate waiting areas. Procedural challenges included the following: nonstandardized triage protocols; unclear role delineation; confusing sample collection procedures; insufficient training in STI examination, testing, and treatment procedures; variable documentation practices; and difficulty ensuring follow-up. Suggested solutions included development of role-specific STI protocols, smart phrases for documentation, improved sample-labeling procedures, prepackaged medications for empiric treatment, and embedded referral pathways to an adolescent care clinic.

**Conclusion:**

Using the TICD framework, this study identified key challenges, such as gaps in training, protocols and transition of care, and corresponding stakeholder-informed solutions to improve adolescent care for STIs in a pediatric ED setting. These findings can guide efforts to implement evidence-based STI practice guidelines in PEDs and general EDs.

## INTRODUCTION

Adolescents and young adults 15 to 24 years of age accounted for almost half of new sexually transmitted infection (STI) cases diagnosed in the United States (U.S.) in 2023.^1^ Although the U.S. Centers for Disease Control and Prevention (CDC) has promulgated guidelines on STI evaluation and treatment, there have been reports of poor compliance with recommended practice in U.S. pediatric emergency departments (PEDs) and general EDs.^2^ Despite often being the initial care clinicians for adolescents and young adults with possible STIs, PEDs and general EDs have demonstrated deficiencies in STI evaluation and treatment, including the following: absent or incomplete documentation of sexual histories with differences by sex; incomplete physical examinations; lack of or incomplete STI testing (including lack of HIV testing) with differences by sex; lack of pregnancy testing; disparate documentation and performance of STI or pregnancy testing by race; STI treatment provision not in accordance with guidelines; disparate STI treatment by sex, Hispanic ethnicity, or race; incomplete or missing STI-related discharge instructions with differences by sex; and lack of referral to outpatient follow-up care.^3^–^25^ Understanding the challenges that prevent healthcare professionals from providing STI care to adolescents and young adults according to CDC guidelines is a necessary first step in the pathway to improve STI care among adolescents and young adults.

We sought to develop, deploy, and evaluate an implementation strategy plan designed to improve the delivery of STI care at a U.S. urban academic center PED. The evidenced-based practices we implemented were the 2021 CDC STI Treatment Guidelines.^2^ To inform this implementation strategy plan, we interviewed key nursing and physician stakeholders involved in the care of PED patients with possible STIs. The goal was to identify challenges and suggested solutions related to conducting six key steps in ED STI care (triage and initial assessment, medical history taking and documentation, physical examination and documentation, STI testing, empiric treatment, and follow-up arrangements) in accordance with CDC guidelines. This formative qualitative study was guided by this research question: What barriers and stakeholder-informed solutions do PED nursing and physician staff identify during each of the six stages of STI care delivery? The objective of this study was to elucidate and characterize the challenges and accompanying suggested solutions to overcome those challenges in delivering STI guideline-concordant care in this PED, as reported by PED nursing and physician staff. Our ultimate objective was to use the findings to inform the development of an implementation strategy plan to improve delivery of guideline-concordant STI care in the PED.

## METHODS

### Study Design, Setting, and Research Team

This investigation was conducted as part of a three-phase (formative, implementation and evaluation phases) larger implementation project designed to improve STI care in a U.S. PED. The larger project aimed to develop, deploy, and evaluate an implementation strategy plan to increase the delivery of STI care in the PED concordant with the 2021 CDC STI Treatment Guidelines. The evidence-based practices targeted for implementation in this plan were these guidelines. This paper reports only the formative qualitative phase of the project, which involved semi-structured interviews with PED nursing and physician stakeholders to identify challenges, facilitators, and suggested solutions related to guideline-concordant STI care delivery in the PED.

**Population Health Research Capsule**

What do we already know about this issue?

*Pediatric emergency departments (PEDs) and general EDs do not consistently deliver sexually transmitted infection (STI) care that aligns with clinical guidelines*.

What was the research question?

*What do PED nurses and physicians identify as the challenges and solutions to providing guideline-concordant care for patients with a possible STI?*

What was the major finding of the study?

*Nurses and physicians offered actionable solutions to address impediments to providing care to STI patients*.

How does this improve population health?

*Suggested solutions to challenges in providing STI care in the PED that could assist in reducing the spread and morbidity of these infections*.

The qualitative research design was a case study of the STI care processes in this PED, in reference to the 2021 CDC STI Treatment Guidelines.^2^ The qualitative research paradigm was pragmatic, such that although the interviews were designed to address Tailored Implementation in Chronic Diseases (TICD) implementation determinants, the semi-structured interviews were flexible to permit new information to be explored as it arose. The study was informed by the TICD framework and organized according to the six sequential workflow stages of ED STI care: triage and initial assessment; history-taking; physical examination; testing; treatment; and follow-up.^26^

The PED for this project was situated at the Mount Sinai Hospital, a quaternary care, urban, academic center in New York City that hosts emergency medicine (EM), pediatrics, and family medicine residencies, as well as a pediatric emergency medicine (PEM) fellowship. The PED clinical team is composed of nine PEM attending physicians, three PEM fellows, and 26 full- and part-time nurses. Emergency medicine (EM) residents from two separate residency programs, pediatrics residents from one residency program, and family medicine residents from one residency program (all of which are affiliated with the medical school) provide additional medical staff coverage. The study was approved by the medical school’s institutional review board. We followed the Standards for Reporting Qualitative Research checklist to ensure transparency and rigor in reporting.^27^ The research team was comprised of three emergency physicians (including a PEM-fellowship trained PEM physician and two adult emergency physicians trained in implementation science methodology), an adolescent health physician, and a survey methodologist.

### Determinants Framework and Interview Guide Development

To identify the relevant determinants of implementing improved processes for STI care, we employed the TICD framework for this project. The research team reviewed all TICD domains and selected those most applicable to the delivery of evidence-based STI care in the PED. Each selected determinant was prioritized by the research team based on its importance and feasibility. The resultant list of prioritized determinants was paired with an exploration process—research reviews, expert consultations (eg, with pharmacists or information technologists (IT)), or stakeholder interviews. This paper reports the work from the stakeholder interviews.

For the stakeholder interviews, we developed interview guides to explore the prioritized TICD determinants in context of the goals of the project. We created the semi-structured interview scripts for this investigation, which included open-ended questions and prompts to elaborate on the responses. The guides were structured around the usual six successive workflow stages of STI care in the PED: 1) triage and initial assessment; 2) obtaining the patient history; 3) conducting the physical examination; 4) testing; 5) treatment; and 6) arranging follow-up. Questions were designed to elicit responses about standard practices, barriers, facilitators, and potential solutions to improve STI care. Separate guides were developed for physicians and nurses. Interview scripts are available in the Supplement 1 (nurses) and Supplement 2 (physicians).

### Participant Recruitment and Interview Procedures

Using stratified purposive sampling, we recruited stakeholders targeting at least five participants per stratum (eg, attending physicians, residents, nurses) in accordance with qualitative research best practices to achieve saturation.^28^ Residents were eligible only if they had completed at least one month of PED service. At least one resident from each postgraduate year was recruited to permit a range of perspectives based on years of experience. Family medicine residents were not included in the interviews because they perform few clinical shifts in the PED, in contrast to EM and pediatrics residents. The final stakeholder group was comprised of six EM residents, six pediatrics residents, six PEM faculty, three PEM fellows (the entire complement), and six PED nurses. Interviewed EM and pediatrics residents range between first year residents to chief residents, who had completed at least one month of PED service. Interviewed faculty members completed a PEM fellowship, and had been with the department for at least three years. The final stakeholder group had 19 females, eight males; 21 identified as White, five identified as Asian, one identified as Hispanic.

Participants were recruited via email and announcements at staff meetings. Because recruitment occurred through a combination of direct outreach and open staff-meeting announcements, the total number of eligible individuals who received or viewed recruitment information was not systematically tracked. Therefore, a response rate could not be calculated. We continued recruitment until the targeted clinical roles were represented. All interviews were conducted virtually by the principal investigator and project manager. Participants confirmed their consent to be included in the study at the start of each interview. The interview guides were semi-structured and included open-ended questions and prompts. Interviews were purposely iterative; when participants raised new workflow issues or suggested solutions relevant to subsequent interviews, the interviewer added or modified probes to explore those topics with later participants. The core interview domains remained consistent across interviews. Interviews were audio recorded and transcribed verbatim. Participants received a gift card.

### Interview Transcript Analyses

Transcripts were analyzed using rapid content analysis. Two physician coders not involved in the interviews nor the creation of the interview guide independently summarized responses by stakeholder type. They met afterward and prepared a combined summary of responses categorized by topic, subtopic and sub-subtopic, which is provided in Supplement 3. Discrepancies were resolved through consensus. Challenges and solutions were categorized by TICD determinants and arranged according to the framework of the six stages of the usual flow of patients through the PED for a visit for a possible STI. Responses were summarized by stakeholder type rather than quantified by individual participant frequency. Therefore, stakeholder abbreviations in the supplemental results table indicate that at least one participant from that stakeholder group identified the corresponding challenge or suggested solution.

## RESULTS

The PED nursing and physician staff reported largely structural and process-related challenges when providing STI care. Table 1 summarizes these challenges, along with their suggested solutions to address them. Challenges and solutions are denoted by stakeholder type: nursing staff; pediatrics residents; EM residents; PEM fellows; and PEM attending physicians. The narrative below explains each challenge and corresponding solution, organized primarily by the clinical stage of care, with relevant TICD determinants highlighted. Representative quotes are included throughout to help illustrate participant viewpoints.

### Triage and Initial Assessment

#### Capacity for Organizational Change: Regulations, Rules, and Policies

Nurses noted that there is no PED policy on triaging procedures for possible STI concerns. They also reported difficulties accurately identifying patients presenting with STI-related complaints at triage. Pediatric EM fellows specifically noted the absence of routine triage screening for STI risk, contributing to overlooked cases early in the visit.

### Incentives and Resources: Availability of Necessary Resources

A central issue identified was insufficient patient privacy. Nurses frequently described challenges maintaining patient confidentiality due to limited private examination spaces and difficulty separating patients from family members. As one nurse explained, it can be “difficult when parents don’t want to leave the room” during sensitive conversations. Another nurse pointed out the current practice of “using the gynecology (GYN) room for non-GYN complaints,” which limits access for adolescent patients needing private exams. Both nurses and physicians expressed frustration with inadequate sound-isolated spaces equipped for gynecologic examinations.

### Patient factors: Behavior, Motivation, and Preferences

Nurses described adolescent embarrassment and reluctance to openly discuss sensitive concerns at triage. Medical staff have also noted that patients hesitate to speak about their sexual concerns particularly when seated near younger children who could overhear conversations.

### Suggested Solutions

Pediatrics and EM residents, along with attending physicians, recommended triage protocols to assign private rooms immediately to patients suspected of having an STI. Nurses suggested creating dedicated private spaces specifically for adolescent STI concerns. Attending physicians supported establishing separate waiting areas for guardians. Nurses and pediatrics residents emphasized the importance of considering patient age when assigning rooms to prevent adolescents from feeling uncomfortable around younger children.

### Medical History Taking and Documentation

#### Incentives and Resources: Availability of Necessary Resources

Many participants shared that the lack of private, closed patient examination rooms and thus reliance on curtained rooms significantly hindered privacy, negatively affecting adolescents’ comfort in sharing truthful medical histories. Lack of in-room computer workstations were also reported as hindering physicians from documenting while taking the patient’s medical history; thus, completion of patient records is often delayed and lacks precision. Residents and fellows also mentioned the lack of standardized interview templates tailored to sexual health concerns available within the electronic health record (EHR), resulting in documentation practices that varied across physicians.

#### Incentives and Resources: Information System and Capacity for Organizational Change: Regulations, Rules, and Policies

Participants expressed confusion over documentation standards and privacy laws, specifically regarding confidentiality and Health Insurance Portability and Accountability Act (HIPAA) compliance for minors, causing reluctance to document sensitive details. One pediatrics resident explained concerns over documentation: “If I were seeing a 14-year-old and I didn’t know what was appropriate to document in the chart…I might be hesitant to document those things fully.”

#### Individual Health Professional Factors: Skills Needed

Participants across stakeholder roles cited barriers to sexual history taking due to discomfort with asking these questions, particularly when related to patient-physician/nurse gender discordance. Emergency medicine residents specifically described challenges due to inadequate privacy, noting that “perceived inconsistencies in patient sexual histories” were often secondary to variations in physician interview methods, a point corroborated by attending physicians. Pediatric EM Fellows and residents also mentioned difficulty remembering to ask pertinent questions about sexual health and difficulty remembering details provided by the patients.

#### Professional Interactions: Team Processes

Pediatrics and EM residents and attending physicians noted inefficiencies and patient inconvenience resulting from multiple nurses and physicians conducting similar evaluations sequentially.

#### Suggested Solutions

In addition to the recommendation of placing patients with STI-related complaints in a dedicated room, physicians also proposed routinely reassuring adolescents and guardians that interviewing privately is standard practice. To improve EHR documentation, physicians suggested standardized smart phrases, templates, and checklists. Attending physicians recommended patient self-administered surveys for sexual history taking. Residents suggested hiring scribes and increasing in-room computer availability to aid in timely completion of EHR records, while fellows recommended privacy restrictions within the EHR to protect sensitive patient information from parents/guardians.

### Conducting and Documenting the Physical Examination

#### Capacity for Organizational Change: Regulations, Rules, and Policies

Attending physicians voiced concerns about redundant and repetitive physical examinations being performed by multiple physicians for the same patient (eg, resident, fellow, attending). Knowledge about institutional policies regarding chaperone guidelines also differed among the physicians. Residents expressed that the responsibilities of residents and nurses overlap, thus creating confusion on who will complete a certain task.

#### Individual Health Professional Factors: Skills Needed

Residents voiced concerns regarding their ability to accurately identify findings, such as cervical motion tenderness. Emergency medicine residents expressed that they do not have sufficient experience in performing a physical examination on a patient with a potential STI. Attendings highlighted anxieties around the legal implications of performing and documenting the results from genitourinary examinations for these patients.

#### Incentives and Resources: Availability of Necessary Resources

Physicians emphasized privacy issues during physical examinations, notably due to curtained exam areas. One EM resident noted discomfort: “I feel like if I was in that situation, (as a patient) I’d be uncomfortable knowing that there was just a curtain or even kind of like a screen.” Participants universally described inadequate equipment, specifically the limited availability of gynecologic beds and examination stirrups. Physicians additionally noted logistical issues, notably delayed patient room assignment and difficulties with promptly finding chaperones.

#### Incentives and Resources: Information System

Patient information privacy concerns were also expressed in this section. Most physicians also voiced uncertainty on guardian access to adolescent patients’ EHR. Physicians described the challenges of delayed documentation postexamination, uncertainty regarding patient information privacy, and parent/guardian access to the patient’s EHR.

#### Suggested Solutions

Participants recommended creating at least one dedicated private examination room in the PED exclusively equipped for genitourinary examinations, along with convertible beds and stirrups. Clear assignment of chaperone duties and improved coordination among medical staff were suggested to streamline care. For documentation improvements, attendings recommended using EHR templates and smart phrases with automated flags. One attending physician noted: “If you use one of those templates... it will give you a flag before you send that chart.”

### Sexually Transmitted Infection Testing

#### Individual Health Professional Factors: Skills Needed

Pediatrics and EM residents said that they receive insufficient training on genitourinary sample collection. All physicians reported uncertainty in selecting the appropriate testing kit for certain specimen sources. They also expressed inadequate familiarity with STI testing protocols, particularly trichomonas testing. An EM resident confessed, “I don’t think I’ve ever tested for trich (trichomonas), which is unfortunate, but I also don’t know how we do it.”

#### Capacity for Organizational Change: Regulations, Rules, and Policies

Fellows emphasized that trichomonas was notably absent from standard STI order sets. Nurses said that the urine sample handling process was unclear, which led to cancelled tests.

#### Incentives and Resources: Availability of Resources

Participants described significant delays locating testing supplies and printing patient labels for specimens. Nurses specifically noted confusion about the required sample types (urine, cervical, etc) since they were not displayed clearly in the EHR, and they highlighted challenges scanning patient labels on specimens before laboratory submission. Emergency medicine residents indicated higher risk of sample mislabeling due to limited nurse-only access to label printing. Most participants cited long waits for laboratory results as problematic, requiring patient follow-up after discharge and creating risks related to patient contact and confidentiality.

#### Suggested Solutions

All participants requested additional training on sample collection. They recommended easily accessible “tip sheets” outlining STI test procedures, automating label printing immediately after orders, and clearly indicating sample types in the EHR. They also proposed routine collection of urine samples at triage for adolescents, and saving urine samples for some time in case a new test needed to be performed. Emergency medicine residents suggested standardized methods for conveying STI results (primarily by telephone), verifying patient contact details during PED visits, and documenting clearly whether contact numbers belonged to patients or guardians.

### Sexually Transmitted Infection Empiric Treatment

#### Individual Health Professional Factors: Awareness and Familiarity with the Recommendation

Some physicians described uncertainty on management guidelines for empiric treatment and persistent symptoms despite usual treatment. Nurses specifically highlighted discomfort and unfamiliarity with diluting and administering ceftriaxone with lidocaine.

#### Patient Factors: Behavior, Motivation, and Preferences

Physicians expressed concerns about patient adherence with a 7-day doxycycline course versus single-dose azithromycin, as well as delayed or inability to obtain prescription medications due to patient affordability concerns. Physicians and nurses described adolescent reluctance to accept intramuscular medications, such as ceftriaxone, due to injection-related pain, and patient hesitancy notifying their sexual partners of their STI diagnosis.

#### Suggested Solutions

Physicians recommended creation of easily accessible instructional documents that describe treatment guidelines. Participants proposed training nurses on alternative ceftriaxone administration methods (intravenous or intramuscular mixed with lidocaine). To address medication dispensation challenges, proposed solutions included providing prepackaged medication kits for patients and their partners in the PED and designating a social worker or health educator to assist patients in obtaining outpatient medications from the hospital pharmacy while the patient was in the PED. To encourage partner treatment, attending physicians proposed enabling STI medications to be prescribed through processes not tied to the patient’s EHR.

### Arrangement of Follow-Up Visits

#### Individual Health Professional Factors: Knowledge About Own Practice

Physicians reported that they typically arrange follow-up visits for patients before discharge, but that it is difficult to do so if the patient is discharged outside outpatient clinic hours or if the PED shift is very hectic. If the follow-up visits were not arranged prior to discharge, physicians noted that they were unsure whether the patients would eventually be scheduled for a follow-up visit or had successfully retrieved their medications from their outpatient pharmacy.

#### Incentives and Resources: Information System

Nurses and physicians highlighted inadequate follow-up systems, difficulty contacting patients after discharge, and challenges coordinating care with external primary care physicians. One attending physician stated, “I don’t know if the patients follow up…universally it would be nice to link our patients directly.” Fellows emphasized patient-related barriers such as low health literacy, language issues, and limited support for appointment scheduling. Participants reported that inaccurate patient contact details frequently impeded postdischarge communication. Emergency medicine residents expressed uncertainty whether the referral order was being received by the appropriate outpatient clinic or patients were being contacted for a follow-up visit.

#### Suggested Solutions

Participants suggested reserving follow-up appointments in advance and scheduling visits before patient discharge. Increased use of EHR dot-phrases, business cards, quick response codes, and interdisciplinary team notifications was also proposed. A pediatric EM fellow described a successful approach at another hospital: “They had an appointment scheduler...they would also change the color of the patient on the ED track board, so then we knew that the appointment had been made.” Fellows also stressed the importance of verifying accurate patient phone numbers at the time STI testing was performed.

## DISCUSSION

This investigation identified stage-specific, stakeholder-reported barriers to delivering guideline-concordant STI care for adolescents and young adults in the PED, as well as practical solutions to inform development of a targeted implementation strategy. The challenges were predominantly structural or procedural, encompassing nurse and physician knowledge gaps; physical resource and setting limitations; deficits in information gathering and sharing; system-level barriers to documentation, communication, and follow-up; lack of privacy; and inefficient and unclear workflows and procedures. Such challenges are likely commonly faced at other PEDs and general EDs. However, they were not considered unsurmountable by stakeholders. Pediatric emergency physician and nurse stakeholders were able to suggest numerous valuable solutions that could lead to better STI care. These solutions likely also could be applied to other PEDs and general EDs, with site-specific adaptations. From this work, we were able to develop an implementation strategy plan designed to improve STI care at our PED so that it was concordant with CDC STI treatment guidelines.^29^ We are evaluating the implementation and desired outcomes from the plan’s deployment, which will be shared in a future publication.

Other quality improvement efforts on STI care conducted at other EDs and PEDs have explored several potential solutions thath have demonstrated impact and were echoed in our participants’ proposed solutions. These efforts include the following: enhanced clinician education on STI screening, especially among asymptomatic patients; the use of computerized screening tools (which have been found to double the rate of STI testing among adolescents); self-collection of samples; scheduling follow-up appointments at ED discharge; and sending text message reminders to schedule and attend a follow-up visit.^30^–^34^ System-level interventions, such as improved documentation of confidential adolescent contact information, similarly have facilitated more effective follow-up and communication of test results.^35^ As implemented in some inpatient settings, interventions aimed at normalizing sexual history taking in the ED setting have also led to increased STI screening rates.^36^ Advancing these solutions into deployable implementation strategy plans and assessing their effectiveness is a necessary subsequent step in guiding best practices for STI care in PEDs and general EDs.

## LIMITATIONS

One limitation of this investigation was that it was conducted only at a single teaching hospital, which could limit the external validity of the results to other hospitals. Because this investigation was a qualitative study using purposive sampling, the findings are not intended to estimate the prevalence of these challenges in this PED or across PEDs or general EDs. However, several challenges identified by participants—including limited privacy, lack of standardized workflows, EHR documentation barriers, testing and sample-handling challenges, and difficulty arranging follow-up—are likely applicable to other emergency care settings that provide STI care to adolescents and young adults. Recall and social desirability biases may have influenced participant responses, and inherent biases of the interviewers and analysts could have impacted the results. Strengths of the study include the use of the TICD framework; a structured interview guide based on STI care workflow in the PED; and independence of the data coders and analyzers from the conduct of the interviews and creation of the interview guide.^26^ In addition, the participant sample included a range of clinical roles (nurses, residents, fellows, attendings), thereby enhancing the ability to gain multiple perspectives from those who evaluate and treat patients with a possible STI.

## CONCLUSION

This investigation identified several challenges that disrupt timely, confidential, and guideline-concordant STI care for adolescents and young adults in the pediatric ED, including limited privacy, lack of standardized workflows, unclear team roles, gaps in STI-specific training, variable documentation practices, testing and sample-handling barriers, challenges with empiric treatment and partner management, and difficulty arranging follow-up after discharge. Organizing findings by clinical stage and TICD domain enabled a structured understanding of specific challenges and corresponding solutions, such as EHR modifications, staff training, triage protocols, and workflow redesign, which should be addressed to improve STI care.

## Supplementary Information



## Figures and Tables

**Table 1:** Summary of nurse- and physician-reported suggested solutions to the challenges in providing sexually transmitted infection care in the pediatric emergency department

Stage of PED STI care	TICD determinant domain	Challenges	Solutions	Participant group which reported challenges and/or suggested solutions to the challenges				
				Nurse	Pediatrics resident	Emergency medicine resident	Pediatric emergency medicine fellow	Attending physician
Triage and initial assessment	Workflow, policy, and role clarity	STI screening questions not standardized	Create triage protocol for STI patients	C/S				
		STI risk not assessed at triage	Add STI risk assessment to triage workflow	S			C	
	Resources; patient privacy and engagement	Patient privacy limited during triage	Enhance privacy during rooming and assessment	C/S				
		Patient preparation and communication are difficult	Improve communication and patient preparation workflow	C/S				
History taking and documentation	Resources; patient privacy and engagement	Lack of privacy during sexual history taking	Enhance privacy during history taking		C/S	C/S	C/S	C/S
	Resources; workflow efficiency	Limited time and long waits interfere with history taking	Expand workforce; consider fast-track pathway		C/S	C/S		C
	Patient engagement; communication	Communication problems affect history taking	Enhance communication strategies			C	C	C/S
	EHR/documentation; patient engagement	Self-administered or nonverbal history options underused	Add electronic or paper sexual history tools					C/S
	Workflow, policy, and role clarity	Age-related privacy laws create uncertainty	Clarify adolescent confidentiality policies					C
	EHR and documentation	Documentation problems	Standardize documentation process; improve EHR		C/S	C/S	C/S	C/S
	Clinician knowledge, skills, and comfort	Clinicians forget sexual health questions	Add prompts or structured interview guide		S	C/S	C	S
		Stigma affects STI conversations	Normalize and destigmatize STI care	S	S			C
	Team processes and care coordination	Repetitive questioning by multiple clinicians	Clarify roles; coordinate workflow; reduce redundancy		C/S	C/S		C/S
Physical examination and documentation	Team processes and care coordination	Repetitive patient evaluation across clinicians	Clarify roles and handoffs			S		C/S
	Workflow, policy, and role clarity	Chaperone expectations unclear	Clarify chaperone guidelines	C	C	C		C
	Clinician knowledge, skills, and comfort	Limited clinician experience with GU exam/sample collection	Standardize exam process; train residents		S	C/S	S	
	Resources; patient privacy and engagement	Lack of privacy during examination	Enhance privacy during examination		C/S	C/S	C/S	C/S
	Resources: space, equipment, supplies, and time	Equipment or supplies unavailable/nonfunctional	Improve equipment and supply availability		C/S	C/S	C/S	C/S
	Resources; workflow efficiency	Delays occur during STI-related examination or care	Decrease delays; improve workflow and staffing	C	C/S	C/S	C/S	C/S
	Team communication	Communication problems affect examination process	Improve team communication during exam workflow				C	
	EHR and documentation	Documentation problems	Standardize exam documentation; add EHR reminders		C/S	C/S	C/S	C/S
	EHR and documentation; confidentiality policy	Uncertainty about EHR privacy for adolescents	Train clinicians on adolescent EHR privacy			C/S	C/S	C
	Patient privacy, preferences, and engagement	Patient reluctance to undergo physical examination	Use patient-centered, trauma-informed examination approach	C				C
STI testing	Clinician knowledge, skills, and comfort	Uncertainty about appropriate test selection	Create standardized STI testing algorithm; train clinicians		C/S	C/S	C	C
		Trichomonas testing inconsistent	Include trichomonas guidance in testing protocol		C	C	C	C
		Sample collection technique varies	Standardize collection procedures		C	C/S		
	Patient privacy and communication	STI testing discussions lack discretion in open areas	Protect privacy during testing discussions		C/S			
	Workflow, policy, and role clarity	Sample handling process unclear	Standardize sample handling, labeling, and drop-off	C	C	C/S	C	
		Sample collection problems	Create sample collection workflow and instructions	C/S	C	C		C/S
		Legal concerns about GU documentation	Clarify medicolegal documentation guidance					C/S
	Resources: equipment, supplies, and EHR support	Testing supplies unavailable	Improve testing supply availability	S	S	C/S		C/S
	Resources; EHR and documentation	STI test label-printing delays	Fix label-printing workflow/EHR process	S		C	C	C
	Resources; follow-up/results workflow	Extended wait for STI test results	Improve results workflow and follow-up process		C		C	C
	Resources; lab/testing workflow	Test processing problems	Coordinate with laboratory/testing workflow	C				
Empiric treatment	Clinician awareness and familiarity with recommendations	Abstinence counseling unclear	Add abstinence counseling to treatment guide				C/S	
		Trichomonas undertreatment	Include trichomonas treatment in guidance					C/S
		Persistent symptoms management unclear	Add persistent symptoms pathway					C/S
		Empiric treatment indications unclear	Create empiric treatment algorithm				C/S	
		Medication regimen selection varies	Standardize recommended regimens				C/S	
	Patient engagement; adherence	Treatment adherence challenging	Provide patient-centered treatment education		C	C	C/S	C
	Patient engagement; follow-up systems	Unable to track adherence after discharge	Improve postdischarge tracking/follow-up		C	C		
	Patient engagement; partner management	Partner treatment difficult to ensure	Strengthen partner treatment/EPT pathway		C/S	S	S	C/S
	Patient privacy, preferences, and engagement	Privacy concerns during treatment	Protect privacy during treatment counseling		C	S	C	C/S
	Resources: medication administration and access	Medication access and administration barriers	Coordinate with pharmacy and insurance; revise medication workflow	C/S	S		S	C/S
Follow-up arrangements	Follow-up systems and EHR tracking	Unable to track follow-up after discharge	Streamline follow-up process		C/S	C/S	C	C/S
	Referral processes and interdisciplinary care	Outpatient pediatricians may be uncomfortable treating STIs	Develop interdisciplinary follow-up team			S		C/S
	Referral processes and communication	Referral and communication processes inconsistent	Standardize referral and communication process			C/S	C/S	C/S
	Follow-up systems and referral support	Physicians face barriers making follow-up arrangements	Create systems to support follow-up arrangements		C/S	C	C/S	C
	Patient access and engagement	Patients face barriers arranging follow-up	Assist with appointment scheduling; offer phone follow-up	S	C/S	C/S	C	C/S
	Patient privacy and confidentiality	Follow-up privacy concerns	Use confidential contact and disclosure procedures		C			C
	Follow-up communication and results disclosure	Results disclosure after discharge challenging	Standardize results disclosure workflow					C/S
	EHR and follow-up contact information	Contact information incomplete or inaccurate	Verify contact information in EHR					C/S

*C*, challenges; *EHR*, electronic health record; *GU*, genitourinary; *PED*, pediatric emergency department; *S*, solutions; *STI*, sexually transmitted infection; *TICD*, Tailored Implementation in Chronic Diseases.
