# Supplementary material for: Nurse and Physician Stakeholder Guidance on Improving Sexually Transmitted Infection Care in a Pediatric Emergency Department
Source: West J Emerg Med. 2026 Sep 9;27(5):1305–16. doi: 10.5811/westjem.61866 (PMC13660633; doi:10.5811/westjem.61866)
Supplement: Supplementary file 1 [file wjem-27-1305-s001.docx]

**Supplement 1. Interview Guide for Nurse Stakeholders**

**Nurse Stakeholders**

Thank you for being a part of this study and talking to us today. To recap, the goal of this study is to determine how we can improve the quality of care provided to Mount Sinai Hospital Pediatric Emergency Department patients who possibly have chlamydia, gonorrhea, or trichomonas.

We'd like to hear your thoughts regarding the following six topics:

1. Triaging or Identifying Patients with possible chlamydia, gonorrhea, or trichomonas
2. Preparing the Patient with possible chlamydia, gonorrhea, or trichomonas for a Physical Examination
3. Testing Patients for possible chlamydia, gonorrhea, or trichomonas
4. Providing Treatment for possible chlamydia, gonorrhea, or trichomonas
5. Education/Training of Nurses on Guidelines Processes
6. Communication/Coordination with Laboratory, Medical, and Pharmacy Staff

Following that, we will inquire about any problems with these processes and how they can be improved.

Do you have any questions before we begin?

TRIAGING OR IDENTIFYING PATIENTS WITH POSSIBLE CHLAMYDIA, GONORRHEA, OR TRICHOMONAS

Please tell us how you are (a) triaging or (b) identifying a patient with possible chlamydia, gonorrhea or trichomonas in the Mount Sinai Hospital Pediatric Emergency Department. Please walk us through the steps.

**Purpose: Assess how nurses are triaging or identifying a patient at the Mount Sinai Hospital Pediatric Department with possible chlamydia, gonorrhea, or trichomonas.**

**Determinants:**

- **Awareness and Familiarity**
- **Expected Outcome**
- **Rules, Regulations, and Policies**

1. What problems do you as a nurse encounter when (a) triaging or (b) identifying a patient at the Mount Sinai Hospital Pediatric Emergency Department with possible chlamydia, gonorrhea or trichomonas?
2. How might these problems be resolved? How else could this be improved?

PREPARING THE PATIENT WITH POSSIBLE CHLAMYDIA, GONORRHEA, OR TRICHOMONAS FOR A PHYSICAL EXAMINATION

Please tell us how you as a nurse prepare a patient at the Mount Sinai Hospital Pediatric Emergency Department with possible chlamydia, gonorrhea or trichomonas for a physical examination.

**Purpose: Assess how nurses are preparing a patient at the Mount Sinai Hospital Pediatric Emergency Department with possible chlamydia, gonorrhea, or trichomonas for a physical examination.**

**Determinants:**

- **Skills to Adhere**

1. What problems do you as a nurse encounter when preparing a patient at the Mount Sinai Hospital Pediatric Emergency Department with possible chlamydia, gonorrhea or trichomonas for a physical examination?
2. How might these problems be resolved? How else could this be improved?

TESTING PATIENTS FOR POSSIBLE CHLAMYDIA, GONORRHEA, OR TRICHOMONAS

Please tell us how you as a nurse carry out orders when testing a patient at the Mount Sinai Hospital Pediatric Emergency Department with possible chlamydia, gonorrhea or trichomonas. Please walk us through the steps.

**Purpose: Assess how nurses are carrying out orders when testing a patient at the Mount Sinai Hospital Pediatric Emergency Department with possible chlamydia, gonorrhea, or trichomonas.**

**Determinants:**

- **Knowledge about own practice**
- **Skills to Adhere**
- **Awareness and Familiarity**

1. What problems do you as a nurse have carrying out orders for testing a patient at the Mount Sinai Hospital Pediatric Emergency Department with possible chlamydia, gonorrhea or trichomonas?
   a. How might extragenital testing and patient self-collect swabs be incorporated in the ED workflow?
2. How might these problems be resolved? How else could this be improved?

PROVIDING TREATMENT FOR POSSIBLE CHLAMYDIA, GONORRHEA, OR TRICHOMONAS

Please tell us how you as a nurse carry out orders for treating a patient at the Mount Sinai Hospital Pediatric Emergency Department with possible chlamydia, gonorrhea or trichomonas. Please walk us through the steps. Please tell us about the administration of ceftriaxone.

**Purpose: Assess how nurses are carrying out orders for treating a patient at the Mount Sinai Hospital Pediatric Emergency Department with possible chlamydia, gonorrhea, or trichomonas.**

**Determinants:**

- **Knowledge about own practice**
- **Skills to Adhere**
- **Awareness and Familiarity**

1. What problems do you as a nurse encounter when you carry out orders for treating a patient at the Mount Sinai Hospital Pediatric Emergency Department with possible chlamydia, gonorrhea or trichomonas?
2. How might these problems be resolved? How could this be improved?

EDUCATION/TRAINING OF NURSES ON GUIDELINES PROCESSES

**Purpose: Elicit training suggestions on providing nursing care to a patient at the Mount Sinai Hospital Pediatric Emergency Department with possible chlamydia, gonorrhea, or trichomonas.**

**Determinants:**

- **Skills to Adhere**

1. How would you as a nurse like to be trained on providing nursing care to a patient at the Mount Sinai Hospital Pediatric Emergency Department with possible chlamydia, gonorrhea, or trichomonas?

Prompts:

(a) How is this training being done now?
(b) How would you like training to be done?

**Purpose: Assess access to information resources when providing nursing care to patients at the Mount Sinai Hospital Pediatric Emergency Department with possible chlamydia, gonorrhea, or trichomonas.**

**Determinants:**

- **Information System**

2. How would you as a nurse like to obtain guidance when you have questions on providing nursing care to a patient at the Mount Sinai Hospital Pediatric Emergency Department with possible chlamydia, gonorrhea, or trichomonas when on shift at work?

Prompts:

(a) How are you obtaining this guidance now?
(b) How would you like to obtain this guidance?

**Purpose: Solicit feedback mechanisms on providing nursing care to a patient at the Mount Sinai Hospital Pediatric Emergency Department with possible chlamydia, gonorrhea, or trichomonas.**

**Determinants:**

- **Monitoring Feedback**

3. How would you as a nurse like to get feedback on the quality of providing nursing care to a patient at the Mount Sinai Hospital Pediatric Emergency Department with possible chlamydia, gonorrhea, or trichomonas?

Prompts:

(a) How are you obtaining this feedback now?
(b) How would you like to obtain the feedback?

COMMUNICATION/COORDINATION WITH NURSING STAFF, MEDICAL STAFF, LABORATORY, AND PHARMACY

**Purpose: Solicit advice on improving communication and coordination between nursing and other hospital staff when providing nursing care to a patient at the Mount Sinai Hospital Pediatric Emergency Department with possible chlamydia, gonorrhea, or trichomonas.**

**Determinants:**

- **Communication and Influence**
- **Team Processes**

1. What can be done to improve communication and coordination between nursing staff and other hospital staff for providing nursing care to a patient at the Mount Sinai Hospital Pediatric Emergency Department with possible chlamydia, gonorrhea, or trichomonas?

Prompt/Prompts:

(a) What about coordination? What about communication?
    a. Between nursing staff
    b. Between nursing staff and medical staff
    c. Between nursing staff and pharmacy staff
    d. Between nursing staff and laboratory staff

OVERARCHING/SUMMARY/OTHER ITEMS NOT COVERED ELSEWHERE

**Purpose: Provide an opportunity to discuss any other problems and solutions for providing nursing care to a patient at the Mount Sinai Hospital Pediatric Emergency Department with possible chlamydia, gonorrhea, or trichomonas.**

1. What other problems that we have not already discussed do you as a nurse encounter when caring for a patient at Mount Sinai Hospital Pediatric Emergency Department with possible chlamydia, gonorrhea, or trichomonas?
2. How might these problems be resolved? How could this be improved?
3. If you could develop the perfect system for how the Mount Sinai Pediatric Emergency Department provides nursing care for possible chlamydia, gonorrhea, or trichomonas what would it be?

**Supplement 2. Interview guide for Physician Stakeholders**

**Physician Stakeholders**

Thank you for being a part of this study and talking to us today. To recap, the goal of this study is to determine how we can improve the quality of care provided to Mount Sinai Hospital Pediatric Emergency Department patients who possibly have chlamydia, gonorrhea, or trichomonas.

We'd like to hear your thoughts regarding the following seven topics:

1. Obtaining and Documenting the Patient History
2. Performing and Documenting the Patient Examination
3. Testing for possible chlamydia, gonorrhea, or trichomonas
4. Providing Treatment for possible chlamydia, gonorrhea, or trichomonas
5. Arranging Follow-up Visits for possible chlamydia, gonorrhea, or trichomonas
6. Education/ Training of Physicians on Guidelines Processes
7. Communication/ Coordination with Laboratory, Nursing, Outpatient Providers, and Pharmacy Staff

Following that, we will inquire about any problems with these processes and how they can be improved.

Do you have any questions before we begin?

OBTAINING AND DOCUMENTING THE PATIENT HISTORY

Please tell us how you as a physician are obtaining and documenting the history of a patient at the Mount Sinai Hospital Pediatric Emergency Department with possible chlamydia, gonorrhea or trichomonas. Please walk us through the steps.

**Purpose: Assess problems and solicit suggestions for improving how medical staff obtain the history of a patient at the Mount Sinai Hospital Pediatric Emergency Department with possible chlamydia, gonorrhea, or trichomonas.**

**Determinants:**

- **Information System**
- **Availability of Necessary Resources**

1. What problems do you as a physician encounter when obtaining the history of a patient at the Mount Sinai Hospital Pediatric Emergency Department with possible chlamydia, gonorrhea or trichomonas? We are particularly interested in system-related problems you encounter as a physician, as opposed to patient-related problems.
2. How might these problems be resolved? How could this be improved?

**Purpose: Assess problems and solicit suggestions for improving how medical staff document the history of a patient at the Mount Sinai Hospital Pediatric Emergency Department with possible chlamydia, gonorrhea, or trichomonas.**

**Determinants:**

- **Information System**
- **Availability of Necessary Resources**

3. What problems do you as a physician encounter when documenting the history of a patient at the Mount Sinai Hospital Pediatric Emergency Department with possible chlamydia, gonorrhea or trichomonas?

4. How might these problems be resolved? How could this be improved?

PERFORMING AND DOCUMENTING THE PATIENT EXAMINATION

Please tell us how you as a physician are examining and documenting the examination of a patient at the Mount Sinai Hospital Pediatric Emergency Department with possible chlamydia, gonorrhea or trichomonas. Please walk us through the steps.

**Purpose: Assess problems and solicit suggestions for improving how medical staff examine a patient at the Mount Sinai Hospital Pediatric Emergency Department with possible chlamydia, gonorrhea, or trichomonas.**

**Determinants:**

- **Quality Assurance and Patient Safety**
- **Skills to Adhere**
- **Rules, Regulations, and Policies**

1. What problems do you as a physician encounter when *examining* a patient at the Mount Sinai Hospital Pediatric Emergency Department with possible chlamydia, gonorrhea or trichomonas?
2. How might these problems be resolved? How could this be improved?

**Purpose:** **Assess problems and solicit suggestions for improving how medical staff document an examination of a patient at the Mount Sinai Hospital Pediatric Emergency Department with possible chlamydia, gonorrhea, or trichomonas.**

**Determinants:**

- **Quality Assurance and Patient Safety**
- **Skills to Adhere**
- **Rules, Regulations, and Policies**

3. What problems do you as a physician encounter when *documenting* an examination of a patient at the Mount Sinai Hospital Pediatric Emergency Department with possible chlamydia, gonorrhea or trichomonas?

4. How might these problems be resolved? How could this be improved?

TESTING FOR POSSIBLE CHLAMYDIA, GONORRHEA, OR TRICHOMONAS

Please tell us how you as a physician are testing a patient at the Mount Sinai Hospital Pediatric Emergency Department with possible chlamydia, gonorrhea or trichomonas. What collection methods are you using?

**Purpose: Assess problems and solicit suggestions for improving how medical staff test a patient at the Mount Sinai Hospital Pediatric Emergency Department with possible chlamydia, gonorrhea, or trichomonas.**

**Determinants:**

- **Skills to Adhere**
- **Rules, Regulations, and Policies**

1. What problems do you as a physician encounter when testing a patient at the Mount Sinai Hospital Pediatric Emergency Department with possible chlamydia, gonorrhea or trichomonas?
   a. How about extragenital samples? (i.e. pharyngeal and rectal samples) Can you provide details on how this should be done?
   b. How about patient self-collected samples? How might this be incorporated in the ED workflow?
2. How could these problems be resolved? How else could this be improved?

PROVIDING TREATMENT FOR POSSIBLE CHLAMYDIA, GONORRHEA, OR TRICHOMONAS

Please tell us how you as a physician decide which patients at the Mount Sinai Pediatric Emergency Department to treat empirically with possible chlamydia, gonorrhea, or trichomonas.

Please also tell us how you as a physician decide which patients at the Mount Sinai Hospital Pediatric Emergency Department to test and not treat empirically with possible chlamydia, gonorrhea, or trichomonas.

**Purpose: Assess problems and solicit suggestions for improving how medical staff treat a patient at the Mount Sinai Hospital Pediatric Emergency Department with possible chlamydia, gonorrhea, or trichomonas.**

**Determinants:**

- **Regulations, Rules and Policies**
- **Skills to Adhere**
- **Knowledge of Practice**

1. What problems do you as a physician encounter when treating a patient at the Mount Sinai Hospital Pediatric Emergency Department with possible chlamydia, gonorrhea or trichomonas?
2. How might these problems be resolved? How could this be improved?

ARRANGING FOLLOW-UP VISITS FOR POSSIBLE CHLAMYDIA, GONORRHEA OR TRICHOMONAS

**Purpose: Assess problems and solicit suggestions for improving how medical staff arrange a follow-up visit for a patient at the Mount Sinai Hospital Pediatric Emergency Department with possible chlamydia, gonorrhea, or trichomonas.**

**Determinants:**

- **Physicians Preference**
- **Knowledge of Practice**

Please tell us how you as a physician arrange a follow-up visit for a patient at the Mount Sinai Hospital Pediatric Emergency Department with possible chlamydia, gonorrhea, or trichomonas.

1. What problems do you as a physician encounter when arranging a follow-up visit for a patient at the Mount Sinai Hospital Pediatric Emergency Department with possible chlamydia, gonorrhea or trichomonas?
2. How could these problems be resolved? How else could this be improved?

EDUCATION/TRAINING OF PHYSICIANS ON GUIDELINES PROCESSES

**Purpose: Elicit training suggestions on providing medical care to a patient at the Mount Sinai Hospital Pediatric Emergency Department with possible chlamydia, gonorrhea, or trichomonas.**

**Determinants:**

- **Assistance for Clinicians**
- **Accessibility of Resources**
- **Information Support**

1. How would you as a physician like to be trained on providing medical care to a patient at the Mount Sinai Hospital Pediatric Emergency Department with possible chlamydia, gonorrhea, or trichomonas?

Prompts:

(a) How is this training being done now?
(b) What would you like training to be done?

**Purpose: Assess access to information resources when providing medical care to a patient at the Mount Sinai Hospital Pediatric Emergency Department with possible chlamydia, gonorrhea, or trichomonas.**

**Determinants:**

- **Information System**

2. How would you as a physician like to obtain guidance when you have questions on providing medical care to a patient at the Mount Sinai Hospital Pediatric Emergency Department with possible chlamydia, gonorrhea, or trichomonas when on shift at work?

Prompts:

(a) How are you obtaining this guidance now?
(b) How would you like to obtain this guidance?

**Purpose: Solicit feedback mechanisms on providing medical care to a patient at the Mount Sinai Hospital Pediatric Emergency Department with possible chlamydia, gonorrhea, or trichomonas.**

**Determinants:**

- **Monitoring Feedback**

3. How would you as a physician like to get feedback on their performance on providing medical care to a patient at the Mount Sinai Hospital Pediatric Emergency Department with possible chlamydia, gonorrhea, or trichomonas?

Prompts:

(a) How are you obtaining this feedback now?
(b) How would you like to obtain the feedback?

COMMUNICATION/COORDINATION WITH LABORATORY, NURSING, OUTPATIENT PROVIDERS, AND PHARMACY STAFF

**Purpose: Solicit advice on how to improve communication and coordination between medical staff and other hospital staff when providing medical care to a patient at the Mount Sinai Hospital Pediatric Emergency Department with possible chlamydia, gonorrhea, or trichomonas.**

**Determinants:**

- **Communication and Influence**
- **Team Processes**

1. What can be done to improve communication and coordination between medical staff and other hospital staff when providing medical care to a patient at the Mount Sinai Hospital Pediatric Emergency Department with possible chlamydia, gonorrhea, or trichomonas?

Prompt:

(a) What about coordination? What about communication?
    a. Between medical staff
    b. Between medical staff and nursing staff
    c. Between medical staff and pharmacy staff
    d. Between medical staff and laboratory staff
    e. Between medical staff and outpatient providers

OVERARCHING/SUMMARY/OTHER ITEMS NOT COVERED ELSEWHERE

**Purpose: Provide an opportunity to discuss any other problems and solutions when providing medical care to a patient at the Mount Sinai Hospital Pediatric Emergency Department with possible chlamydia, gonorrhea, or trichomonas.**

1. What other problems that we have not already discussed do you as a physician encounter when caring for a patient at the Mount Sinai Hospital Pediatric Emergency Department with possible chlamydia, gonorrhea, or trichomonas?
2. How could those problems be resolved? How else could this be improved?
3. If you could develop the perfect system for how the Mount Sinai Pediatric Emergency Department provides medical care for possible chlamydia, gonorrhea, or trichomonas what would it be?

**Supplement 3: Categorization of participant responses on challenges to and suggested solutions to overcoming the challenges in delivering STI care in the PED by physician role (pediatrics residents, emergency medicine residents, PEM attending physicians, PEM fellows) and nurses**

**Table A. Pediatrics residents: challenges to and suggested solutions to overcoming the challenges in delivering STI care in the PED**

| **Challenge or solution** | **Topic** | **Subtopics** | **Sub-subtopics** |
| --- | --- | --- | --- |
| **Medical history taking and documentation** | | | |
| Challenges related to medical history taking | Lack of patient privacy | Limited private space availability | No noise barriers in rooms |
|  |  |  | Curtained spaces |
|  |  |  | Difficulty ensuring privacy to  obtain truthful medical history |
|  |  |  | Not enough chairs for physicians |
|  |  | Separating companions (e.g. parents, guardian, partner) from patients | Lack of waiting space for companions (e.g. parents, guardian, partner) |
|  | Stigma around STIs | Phrasing questions to avoid stigma | Asking adolescents about  number of sexual partners |
|  | Limited time availability to take care of patients | Full sexual history takes time |  |
|  | Concerns about the privacy laws  according to patient age |  |  |
|  | Other options to obtain history  are not being used |  |  |
| Solutions to medical history taking challenges | Enhanced triage protocol for STI patients | Triage nurses directing room designations |  |
|  |  | Designate private rooms for patients  with genitourinary complaints |  |
|  | Triage optimization | Improve STI problem identification during triage | Triage nurses assign the  rooms based on STI complaint |
|  | Enhance privacy | Ensure a proper space for taking histories | Designation of waiting room  for the patient's companions |
|  |  |  | Designation of a private area  for the ED provider to sit down |
|  |  | Designate private rooms for  patients with STI complaints |  |
|  |  | Separate companions from patients |  |
|  | Screen for STI concerns during  interview with patient | Inquire about desire to be tested for STIs |  |
|  |  | Alternative ways to screen for STI concerns |  |
|  | Change in workflow | Wait to place patients with genitourinary complaints in a private room instead of immediate placement in non-private area |  |
|  | Destigmatize | Normalize discussions about sexual health | Inform parents/guardians that routine questions will be asked to  every adolescent privately |
| Challenges related to medical history documentation | Documentation Challenges | Incomplete documentation | Prolonged time frame between patient care and documentation |
|  |  |  | Documentation details included by physician depend on who will have access to the medical note (e.g., parent, guardian) |
|  |  |  | Difficulties remembering all  details mentioned by patient |
|  |  |  | New trainees |
|  |  | Documentation challenges of obtaining  complete medical history | Medical history regarding STIs and their treatment is not carried on to later EHR notes |
|  | Lack of privacy | Unclear EHR privacy restrictions regarding  access to My Chart (e.g., parent, guardian) | “Age gap” when patients vs. parents/guardians control access to EHR |
| Solutions to medical history documentation challenges | Standardize the documentation process | Checklists for documentation | Accessible from mobile devices |
|  |  |  | Provided on laminated cards |
|  |  |  | Flow chart symptom checklist |
|  |  |  | Accessible through QR code |
|  |  |  | Automatic transfer of information  from checklist into EHR |
|  |  |  | Patients complete history on devices  which is transmitted to EHR |
|  |  |  | Pop-up reminders on EHR |
|  |  | Prepopulated template in EHR, Smart phrases |  |
|  | Enhance privacy | Clarify Epic Privacy Restrictions | Accessible guidelines on EHR privacy restrictions for parents/guardians |
|  | Accessible computer's locations | Computers in patients' rooms |  |
|  |  | Workstations on wheels |  |
|  | Workforce expansion | Hire medical scribes |  |
| **Conducting and documenting the physical examination** | | | |
| Challenges related to physical examination | Lack of privacy | Limited private space availability | Only one room equipped  for gynecology exams |
|  |  |  | Curtained spaces |
|  |  |  | The gynecology room occasionally  serves as an isolation area |
|  |  |  | No noise barrier in rooms |
|  | Functional equipment and supplies availability | Only one room equipped for gynecology exams | The gynecology room occasionally  serves as an isolation area |
|  |  |  | Potential for missed findings due to  inadequate physical examination |
|  |  | New trainees are unfamiliar  with the supply locations |  |
|  |  | Insufficient availability of clean  gowns, sheets, and linens |  |
|  | Delays in patient care | Difficulties with chaperone availability |  |
|  |  | Difficulties coordinating with attendings to perform genitourinary exam together |  |
| Solutions to physical examination challenges | Functional equipment and supplies availability | Centralized storage space |  |
|  |  | Clearer labeling of supplies |  |
|  |  | Booster chairs for genitourinary exam |  |
|  |  | Detachable stirrups for stretchers |  |
|  | Orientation and training for residents | Inform residents about supplies cabinet |  |
|  | Enhanced triage protocol for STI patients | Triage nurses direct room designations |  |
|  | Enhance privacy | Increase number of private rooms |  |
|  |  | Ensure a proper space for exams | Use a privacy screen |
|  | Standardize the physical examination process | Create a flowchart |  |
|  | Avoid sexualizing language |  |  |
|  | Decrease delays in patient care | Ask patient to put on a gown in advance |  |
|  |  | Use Child Life as chaperones |  |
| Challenges related to physical examination documentation | Documentation challenges | Incomplete documentation | Prolonged time frame between patient  care and documentation |
|  |  |  | Difficulties remembering all details  mentioned by patient |
|  |  | Heterogeneous documentation practices | Heterogeneous genitourinary examination description placement in chart |
|  |  | Pressure to complete templates can  result in inaccuracies |  |
| Solution to physical examination documentation challenges | Standardize the documentation process | Prepopulated template in EHR, Smart phrases |  |
|  |  | Checklists | Distributed on laminated cards |
|  |  |  | Accessible from devices |
|  |  | More inclusive smart block in physical examination section |  |
|  |  | Have a space for free text in physical examination section |  |
| **STI testing** | | | |
| Challenges related to testing for STI | Unclear sample handling process | Unclear sample labeling process |  |
|  |  | Unclear location where to send samples |  |
|  | Uncertainty regarding appropriate test selection | Several different tests |  |
|  |  | Several different types of swabs |  |
|  | Infrequent testing for trichomonas | Lack of knowledge about  trichomonas symptoms | Uncertainty regarding appropriate  test selection |
|  |  |  | Lack of knowledge about how to test |
|  |  |  | Unclear trichomonas testing indication |
|  |  | Misconceptions of trichomonas  infection prevalence |  |
|  | Extended wait for results | Laboratory-related challenges |  |
|  |  | Lack of point-of-care testing |  |
|  | Insufficient experience with physical examination and sample collection | Insufficient genitourinary and  gynecology training | Infrequent swab collections |
|  | Lack of discretion in open areas when  discussing ordered tests |  |  |
|  | Sample collection challenges | Concerns about patient comfort |  |
|  | Variability in sample collection techniques | Inter-physician variability in testing |  |
| Solutions to challenges related to testing for STI | Offer self-collection of samples | Consider vaginal and rectal samples |  |
|  | Standardize testing process | Create a flow sheet with instructions |  |
|  |  | Prepopulated template in EHR, Smart phrases | Include testing suggestions on Smart phrases |
|  |  | Request urine sample from all adolescents for possible STI testing at triage |  |
|  | Orientation and training for residents | Inform all staff about supplies cabinet |  |
|  |  | More genitourinary and gynecology training |  |
|  | Functional equipment and supplies availability | Microscope in the PED to check for microorganisms in the samples |  |
|  | Testing type reminders in EHR | Pop-up in EHR suggesting testing for trichomonas when gonorrhea and chlamydia testing are ordered |  |
|  | Destigmatize STI testing | Normalize broad-based STI screening |  |
| **STI empiric treatment** | | | |
| Challenges related to STI treatment | Lack of patient privacy | Confidentiality concerns | Insurance contacting parents/guardians |
|  |  |  | Parents/guardians having access to EHR |
|  |  |  | Unclear if parents/guardians can access MyChart notifications |
|  |  |  | Parents’/guardians’ phone number listed on EHR without labeling as such |
|  |  | Unclear EHR privacy restrictions regarding  access to My Chart (e.g., parent, guardian) | “Age gap” when patients vs. parents/guardians control access to EHR |
|  | Problems adhering to treatment | Access to medications | Pharmacy accessibility problems |
|  |  |  | Insurance coverage limitations |
|  |  |  | Financial concerns |
|  |  |  | Patient inability to retrieve  medications from pharmacies without parent/guardian |
|  |  | Tolerance to medication regimen |  |
|  |  | Inability to provide full medication regimen  to patient directly from the ED |  |
|  | Inability to keep track of treatment  adherence post-discharge |  |  |
|  | Difficulties ensuring partner treatment |  |  |
|  | Results disclosure challenges | Difficulty contacting patients | Incorrect contact information |
|  |  |  | Not every patient has access to MyChart |
|  |  | Patient comprehension of treatment |  |
| Solutions to challenges related to STI treatment | Functional equipment and supplies availability | Microscope in the ED to check for microorganisms in the samples | Point-of-care testing |
|  | Expedite partner's diagnosis and treatment | Counseling on how to disclose  diagnosis to partner |  |
|  | Expedite partner treatment | Partner referral to the ED when  notified by patient |  |
|  | Expedite patient's treatment | Provide medication from ED |  |
|  |  | Prefer single dose regimens |  |
|  |  |  |  |
|  | Coordinate with pharmacy | Check availability of medication with  pharmacy before ordering |  |
|  | Coordinate with insurance | Check insurance coverage before ordering |  |
|  | Grant patients' digital access to their EHR |  |  |
|  | Correct documentation of contact information | Differentiate patients' personal phone number from parents’/guardians’ number in EHR |  |
| **Arrangement of follow-up visits** | | | |
| Challenges related to arranging a follow-up | Inability to keep track of patient  follow-up post-discharge | Not knowing about what happens  after the patient leaves the PED |  |
|  | Barriers for physicians to assist in  follow-up arrangements | Assistance to arrange a follow-up depends  on factors external to PED | Day and time in which patient visited the PED affects ability to arrange follow-up |
|  |  |  | How busy the shift is |
|  | Barriers for patients to arrange follow-ups | Relying on patients to arrange  follow-up by themselves |  |
|  | Lack of privacy | Parents/guardians questions about the reason  why patient needs further care |  |
| Solutions to challenges related to arranging a follow-up | Systems for physicians to assist in  follow-up arrangements | Special follow-up appointment slots for  patients discharged from PED |  |
|  |  | Information available about walk-in clinics |  |
|  | Assistance for patients to arrange  an appointment | Administrative staff to schedule appointments |  |
|  | Streamlined follow-up process  while patient is in PED | Expedited follow-up at the PED itself |  |
|  |  | Interdisciplinary follow-up team |  |
|  | Patient-centered education | Provide information about the AHC | Smart phrase in EHR with AHC information |
|  |  |  | Business cards with AHC information |
|  |  |  | QR codes linked to AHC information |
|  |  |  | Emphasize that AHC provides free care |
|  | Telephonic follow-up post-discharge | Confirm medication adherence with patient |  |
|  |  | Inquire about missed appointments |  |

**Table B. Emergency medicine residents: challenges to and suggested solutions to overcoming the challenges in delivering STI care in the PED**

| **Challenge or solution** | **Topics** | **Subtopics** | **Sub-subtopics** |
| --- | --- | --- | --- |
| **Medical history taking and documentation** | | | |
| Challenges related to medical history taking | Lack of privacy | Separating companions (e.g., parents,  guardian, partner) from patients | Reluctance of the parent/guardian to  separate from patient |
|  |  | Limited private space availability | No noise barrier in rooms |
|  |  |  | Curtained spaces |
|  |  | Crowding in the PED |  |
|  |  | Inadequate room designation for patients with genitourinary complaints |  |
|  | Communication challenges | Inconsistent sexual history responses  based on questioning method | Adolescent unawareness of sexual health |
|  |  | Physician discomfort with discussing  sexual health-related topics |  |
|  |  | Concerns about patient's comfort | Patient-physician gender concordance |
|  |  | Workflow coordination with healthcare staff | Interruptions by other team members |
|  | Physicians forgetting to ask pertinent  questions about sexual health |  |  |
|  | Long wait times | Patients feel impatient |  |
|  |  | Rushed physicians |  |
|  |  | Addressing primary care-like complaints  in the PED extends wait times |  |
|  | Redundancy and repetition in  multi-provider patient evaluation | Patients could feel confused |  |
| Solutions to medical history taking challenges | Change in workflow | Wait to place patients with genitourinary complaints in a private room |  |
|  | Screen for STI concerns during  interview with patient | Alternative ways to screen for STI concerns | Provide a self-administered survey for patients |
|  |  |  | Patient self-completes survey demographic characteristics and risk factors |
|  |  |  | Include diverse gender identities  and sexual orientations |
|  | Enhance privacy | Ensure a proper space for taking histories | Increase number of private rooms |
|  |  | Move patient to a less crowded area |  |
|  | Create fast-track for low acuity conditions |  |  |
|  | Enhanced triage protocol for STI patients | Designate private rooms for patients with genitourinary complaints |  |
|  | Workflow coordination with healthcare staff | All team members evaluate  patient at the same time |  |
| Challenges related to medical history documentation | Documentation challenges | Incomplete documentation | Easier to use a general over a complaint-specific EHR template |
|  |  |  | Difficulties deciding what information to include/exclude in the EHR |
|  |  |  | Difficulties remembering all details  mentioned by patient |
|  |  |  | Concerns about legal implications of  what it is documented |
|  |  |  | Prolonged time frame between patient  care and documentation |
|  |  |  | Paraphrasing could end up in lost details |
|  | Lack of privacy | Unclear EHR privacy restrictions regarding  access to My Chart (e.g., parent, guardian) | “Age gap” when patients vs. parents/guardians control access to EHR |
| Solutions to medical history documentation challenges | Standardize the documentation process | Prepopulated template in EHR, Smart phrase | Include STI section in review of systems |
|  |  | Checklists | Automatic transfer of information from external checklist into EHR |
|  |  |  | Provide directly to patients on  mobile device to complete |
|  |  |  | Provide directly to patients  on paper to complete |
|  |  |  |  |
|  | EHR improvements | Create extra space for  sexual history documentation |  |
|  | Orientation and training for residents | Training on documentation practices to handle potentially legal sensitive information |  |
|  | Accessible computer locations |  |  |
|  | Workforce expansion | Hire medical scribes |  |
| **Conducting and documenting the physical examination** | | | |
| Challenges related to physical examination | Lack of functional equipment and  supplies availability | Supply stands are often unavailable | Use of improper objects to put supplies on |
|  | Insufficient experience for physical examination and sample collection | Insufficient genitourinary and  gynecology training | Genitourinary/gynecology training specifically for pediatric population |
|  | Lack of privacy | Separating companions from patients |  |
|  |  | Limited private space availability | Only one room equipped for  gynecology exams |
|  |  |  | Curtained spaces |
|  |  |  | Dysfunctional curtains |
|  | Delays in patient care | Difficulties with chaperone availability | Inadequate assignment of duties |
|  |  |  | Reluctance of nursing and ancillary staff to serve as chaperones |
|  |  |  | Child Life staff cannot chaperone |
| Solutions to physical examination challenges | Workforce expansion | Hire more PED techs |  |
|  | Clarify roles | Chaperone duty distribution among staff | Assign chaperoning duties to techs |
|  |  |  | Assign chaperoning duties to residents |
|  |  |  | Assign chaperoning duties to attending physicians, if available |
|  | Enhanced triage protocol for STI patients | Triage nurses directing room designations |  |
|  | Orientation and training for residents | More genitourinary and gynecology training | Genitourinary/gynecology training specifically for pediatric population |
|  | Functional equipment and supplies availability | Booster chairs for genitourinary exam |  |
|  | Enhance privacy | Ensure a proper space to perform  the physical examination | Utilize a space in the adult ED |
|  |  |  | Use a privacy screen |
|  |  |  | Consider using partially walled areas |
|  |  |  | Reconfigure existing private rooms for patients with genitourinary complaints |
|  | Communication enhancement | Coordinate with staff about knocking before opening curtains/privacy screens |  |
| Challenges related to physical examination documentation | Documentation challenges | Incomplete documentation | Uncertainty about which items to  include in the EHR |
|  |  |  | Prolonged time frame between patient care and documentation |
|  |  |  | Overlooking the documentation  of chaperone presence |
|  |  |  |  |
|  |  | Lack of details in dermatologic descriptions |  |
|  |  | Lack of confidence in reporting certain findings | Documentation of cervical motion tenderness |
|  | Functional equipment and supplies availability | Insufficient workstations |  |
|  | Lack of privacy | Unclear EHR privacy restrictions regarding  access to My Chart (e.g., parent, guardian) | “Age gap” when patients vs. parents/guardians control access to EHR |
| Solution to physical examination documentation challenges | Orientation and training for residents | Training on best documentation practices | Documentation of pictures |
|  |  |  |  |
|  | Standardize the documentation process | Prepopulated template in EHR, Smart phrases |  |
|  |  | Checklists | Provided directly to patients on device |
|  |  | EHR improvements | Create section to document chaperone |
| **STI testing** | | | |
| Challenges related to testing for STI | Infrequent testing for trichomonas | No trichomonas test availability | Unavailability of wet mounts |
|  |  | Lack of knowledge about trichomonas testing | Uncertainty regarding appropriate  STI test selection |
|  | Functional equipment and supplies availability | Unclear supplies locations | Lack of pH paper for vaginal fluids testing |
|  | Uncertainty regarding appropriate test selection | Several different STI tests |  |
|  |  | Several different types of swabs |  |
|  | Unclear sample handling process | Unclear duty assignment | Unclear sample labeling process |
|  | Sample collection challenges | Concerns about patient's comfort |  |
|  | Variability in sample collection techniques | Inter-physician variability | Difficulty choosing between different collection methods and samples |
|  | Delays on labels printing | Labels can only be printed by nurses | Label printing depends on nurses' workflow |
|  |  |  | Risk of mislabeling or losing samples |
| Solutions to challenges related to testing for STI | Standardize testing process | Create STI order set | Order set offering recommendations  for what tests to order |
|  |  |  | Categorize order set by gender, partner  and HIV status |
|  | Functional equipment and supplies availability | More clear labels | Label boxes where STI swabs are stored |
|  |  |  | Guidance diagram in shelf or cabinet |
|  | Self-sample collection for screening | Specify the reason when not considered |  |
|  | Orientation and training for residents | More genitourinary and gynecology training | Training on extragenital testing |
|  |  |  | Sample handling training |
|  | Enhance privacy | Clarify EHR Privacy Restrictions |  |
|  | Designate a location to drop samples off | Plastic container to drop samples off |  |
|  | Standardize labeling process | Labels printed automatically after ordering |  |
|  |  |  |  |
|  |  | Enable residents to print labels |  |
| **STI empiric treatment** | | | |
| Challenges related to STI treatment | Problems adhering to treatment | Lack of access to medications | Patient inability to retrieve  medications from pharmacies  without parent/guardian |
|  |  |  | Medication collection challenges due to access, lack of transportation |
|  |  |  | Financial concerns |
|  |  |  | Unknown preferred pharmacy |
|  |  | Difficulty completing a longer medication course |  |
|  |  | Concerns about refusal of  certain medication administration routes | Reluctance to receive injections |
|  |  | Limited understanding of the medication dispensation process in the PED |  |
|  | Inability to keep track of treatment  adherence post-discharge |  |  |
| Solutions to challenges related to STI treatment | Standardize the documentation process | Create STI order set | Order set offering recommendations for medications to order |
|  | Respect patient's privacy | Disclose results by phone instead of EHR |  |
|  | Expedite patient's treatment in PED | Provide medications from the PED | Provide full course of medications for free |
|  |  |  |  |
|  | Communication enhancement | Standardize communication method | Provide a dedicated phone line  for troubleshooting |
| **Arrangement of follow-up visits** | | | |
| Challenges related to arranging a follow-up | Inability to keep track of patient  follow-up post-discharge | Not knowing about what happens  after the patient leaves the ED | Unclear information about  referral system effectiveness |
|  |  |  | Unfamiliarity with referral system |
|  |  |  |  |
|  | Barriers for physicians to assist in  follow-up arrangements | Lack of mechanisms for physicians  to arrange follow-ups |  |
|  | Barriers for patients to arrange follow-ups | Relying on patients to arrange  follow-up by themselves |  |
| Solutions related to arranging a follow-up | Assistance for patients to  arrange an appointment |  |  |
|  | Streamlined follow-up process at ED | Expedited follow-up at the ED | Walk-in center |
|  | Interdisciplinary follow-up team | Send automatic notifications  of PED visit for STI to the AHC |  |
|  | Patient-centered education | Sexual health handouts | Handouts editable by physicians  during discharge |
|  | Communication enhancement | Standardize communication methods | Provide a dedicated phone line for troubleshooting post-PED visit |
|  | Standardize referral process | Clarify criteria for referrals |  |
|  |  | Develop a referral plan for patients whose primary care providers are not part  of the Sinai system |  |

**Table C. Pediatric emergency medicine attending physicians: challenges to and suggested solutions to overcoming the challenges in delivering STI care in the PED**

| **Challenge or solution** | **Topics** | **Subtopics** | **Sub-subtopics** |
| --- | --- | --- | --- |
| **Medical history taking and documentation** | | | |
| Challenges related to medical history taking | Communication challenges | Inconsistent sexual history responses based  on questioning method | Inter-interviewer variability |
|  |  |  | Adolescents have magical thinking |
|  |  | Language barriers | Limited translation devices |
|  | Lack of privacy | Limited private space availability | No noise barrier in rooms |
|  |  |  | Curtained spaces |
|  |  | Crowding in the PED |  |
|  |  | Separating companions (e.g. parents, guardian, partner) from patients | Reluctance of the parent to  separate from patient |
|  |  | Confidentiality concerns | Patient's confidentiality concerns |
| Solutions to medical history taking challenges | Screen for STI concerns during  interview with patient | Alternative ways to screen for STI concerns | Provide a self-administered survey for patients |
|  | Enhance communication | Use translators |  |
|  |  | Translator app |  |
|  | Enhance privacy | Ensure a proper space to  obtain the patient history | Designation of waiting room for the  patient's companions |
|  |  |  | Use Child Life private office |
|  |  |  | Designate private rooms for patients with genitourinary complaints |
|  |  | Inform parents that routine questions will be asked to every adolescent privately |  |
|  |  | Reassure patient that answers  will be kept confidential |  |
|  | Orientation and training for residents | More genitourinary and gynecology training | Training focused on medical history taking |
|  | Change workflow | Wait to place patients with genitourinary complaints in a private room instead of immediate placement in non-private area | Use the room exclusively for physical examinations, taking turns accordingly |
| Challenges related to medical history documentation | Documentation challenges | Incomplete documentation | Dyspareunia is not in the review  of systems checklist |
|  |  |  | Prolonged time frame between patient care and documentation |
|  |  |  | Difficulties remembering all details  mentioned by patient |
|  |  |  | Documentation details depend on who will have access to the EHR |
|  |  | Limited knowledge about how to use the  history of present illness templates |  |
|  |  | Pressure to complete templates can  result in inaccuracies | Important information could be missed  if it is not in the Smart phrase |
|  |  | Heterogeneous documentation practices | Inter-trainee variability |
|  | Lack of privacy | Unclear EHR privacy restrictions of  access to My Chart | Cares Act lead to immediate  sharing of the notes |
|  |  |  | “Age gap” when patients vs. parents/guardians control access to EHR |
|  |  | Confidentiality concerns | Parents/guardians access to EHR |
| Solutions to Medical history documentation challenges | Workflow coordination with healthcare staff | All team members evaluate  patient at the same time |  |
|  | Standardize the documentation process | Checklist | Provide directly to patients on  mobile device to complete |
|  |  |  | Automatic transfer of information from external checklist into the EHR |
|  |  |  | Accessible from mobile devices |
|  |  |  | Distribute laminated cards |
|  |  | Prepopulated template in EHR, Smart phrase |  |
|  | Enhance privacy | Use EPIC privacy restrictions to prevent guardians to access certain sensitive notes | "Hide the notes" option |
|  |  |  | Pop-up reminder to modify privacy settings based on patient's diagnosis |
|  | Orientation and training for residents | More genitourinary and gynecology training | Training focused on medical history taking |
| **Conducting and documenting the physical examination** | | | |
| Challenges related to physical examination | Delays in patient care | Difficulties with chaperone availability |  |
|  |  | Waiting for patients to put on a gown |  |
|  | Redundancy and repetition in  multi-provider patient evaluation | Workflow coordination with healthcare staff |  |
|  | Lack of privacy | Limited space availability | Only one room equipped  for gynecology exams |
|  |  |  | Curtained spaces |
|  |  |  | The gynecology room occasionally  serves as an isolation area |
|  |  | Separating companions (e.g. parents, guardian, partner) from patients |  |
|  | Reluctance of patients  to be physically examined |  |  |
|  | Functional equipment and supplies availability | Limited access to linen cart |  |
|  |  | Gynecological bed not properly functioning |  |
| Solutions to physical examination challenges | Clarify roles | Chaperone duty distribution among staff | Assign chaperoning duties to Child Life staff |
|  | Workflow coordination with healthcare staff | All team members evaluate  patient at the same time |  |
|  | Communication enhancement | Counsel patient about  revealing STI concern to parent/guardian |  |
|  | Enhance privacy | Ensure a proper space to  conduct the physical exam | Utilize a space in the adult ED |
|  |  |  | Use a privacy screen |
|  |  |  | Increase number of rooms equipped  for gynecology exams |
|  |  |  | Designate a specific room for procedures |
|  |  |  | Fix curtains and ensure they close fully |
|  |  | Room designation changes | Designate private rooms for patients with genitourinary complaints |
|  | Functional equipment and supplies availability | Use convertible chairs from lower acuity area  for gynecology exams |  |
|  |  | Booster chairs for genitourinary exam |  |
|  |  | Stocking gynecology space with linen sets |  |
|  | Change in workflow | Wait to place patients with genitourinary complaints in a private room | Use the room exclusively for physical examinations, taking turns accordingly |
|  | Decrease delays in patient care | Ask patient to put on a gown in advance | Offer gown and sheet on  patient arrival in room |
|  | Enhanced triage protocol for STI patients | Triage nurses directing room designations |  |
| Challenges related to physical examination documentation | Lack of privacy | Unclear EHR privacy restrictions of  access to My Chart | Healthcare providers lack knowledge on how to use EHR’s privacy mechanisms. |
|  | Documentation challenges | Incomplete documentation | Poor coordination between residents and attendings in documenting  the physical examination |
|  |  |  | The resident forgets to update the note after the physical examination |
|  |  |  | Prolonged time frame between  patient care and documentation |
|  |  |  | Inadequately detailed description  of physical findings |
|  |  | Heterogeneous documentation practices | Heterogeneous genitourinary examination description placement in EHR |
| Solution to physical examination documentation challenges | Standardize the documentation process | Prepopulated template in EHR, Smart phrase | Design template according to age categories |
|  |  | Checklists | Pop-up alert when chart is incomplete |
|  |  |  | Mandatory question-based checkboxes |
|  |  | Emphasize every resident's responsibility to write notes unless instructed otherwise by attending | Notify chief residents through email about changes in documentation rules |
|  |  | Attendings document pelvic examinations  they've witnessed |  |
|  | Reminders in EHR | Pop-up alert when chart is incomplete |  |
| **STI testing** | | | |
| Challenges related to testing for STI | Functional equipment and supplies availability | Lack of microscopes |  |
|  | Infrequent testing for trichomonas | No trichomonas test availability | Unavailability of wet mounts |
|  |  | Lack of knowledge about Trichomonas testing |  |
|  | Concerns about legal implications of genitourinary examination |  |  |
|  | Extended wait for results | Lack of point-of-care testing |  |
|  | Uncertainty regarding  appropriate test selection | Several different tests |  |
|  |  | Several different types of swabs |  |
|  | Sample collection challenges | Inadequate urine sample handling | Incorrect filling of urine sample tubes  leads to cancelled tests |
|  |  |  | Concerns about clean urine catch having a lower sensitivity for chlamydia and gonorrhea |
|  | Delays on labels printing |  |  |
| Solutions to challenges related to testing for STI | Legal precautions | Use the ultrasound probe instead of fingers to avoid touching patient's genitals |  |
|  |  | Record video clips during physical  examination for evidence |  |
|  |  | Avoid bimanual exams on younger patients |  |
|  | Instructional documents for  procedural guidance | Physical guidance documents | Short tip sheet |
|  | Orientation and training for nurses | More genitourinary and gynecology training | Sample handling training |
|  | Functional equipment and supplies availability | Microscope in the PED to check for microorganisms in the samples |  |
|  | Clarify how to order STI tests in EHR |  |  |
|  | Standardize testing process | More clear labels | Label boxes where STI swabs are stored |
|  |  | Establish a clear protocol on  STI testing in the PED |  |
|  |  | Universal testing | Request urine sample  from all adolescents at triage |
| **STI empiric treatment** | | | |
| Challenges related to STI treatment | Problems adhering to treatment | Intolerance to medication regimen |  |
|  |  | Inability to provide full medication  regimen to patient from the PED |  |
|  |  | Difficulty completing a longer medication course |  |
|  | Trichomonas infection is undertreated | Lack of trichomonas testing |  |
|  | Unclear management of patients with persistent symptoms despite usual treatment |  |  |
|  | Results disclosure challenges | Difficulty contacting patients post-PED visit | Incorrect contact information |
|  | Lack of privacy | Separating companions (e.g. parents, guardian, partner) from patients |  |
|  | Concerns about refusal of certain  Medication administration routes | Reluctance to receive injections |  |
|  | Difficulties ensuring partner treatment | Reluctance of patients to reveal information to contact their partners |  |
| Solutions to challenges related to STI treatment | Assistance for patients to medication access through hospital pharmacy | Access to a social worker or educator |  |
|  | Functional equipment and supplies availability | Access to medications from the PED | On-site pharmacy |
|  | Expedite patient's treatment | Provide medications for extra  days to give to partners |  |
|  | Enhance privacy | Separate companions from patients | Give discharge instructions in a different room |
|  |  |  | Provide treatment in a different room |
|  |  | Give only general discharge  instructions to parents/guardians |  |
|  | Correct documentation of contact information | Differentiate patients' personal phone number from parents’/guardians’ number in EHR |  |
|  | Expedite partner treatment | Enable prescriptions outside of EHR system |  |
|  |  | Enable prescriptions outside of a patient's chart |  |
|  | Standardize the documentation process | Create STI order set | Order set offering recommendations  for medications to order |
| **Arrangement of follow-up visits** | | | |
| Challenges related to arranging a follow-up | Some private pediatricians are  not comfortable treating STIs | Provider feeling uncomfortable discussing  sexual health-related topics |  |
|  |  | Some private pediatricians' offices are not set up for genitourinary exams |  |
|  | Barriers for physicians to assist in  follow-up arrangements | Lack of mechanisms for physicians to  arrange follow-up |  |
|  |  | Problems deciding the  appropriate referral specialty |  |
|  | Lack of privacy | Confidentiality concerns | Parents’/guardians’ phone number listed in EHR without labeling as such |
|  | Inability to keep track of patient  follow-up post-discharge | Not knowing about what happens after  patient leaves the PED | Difficulty ensuring follow-up  appointment attendance |
|  | Results disclosure challenges | Difficulty contacting patients | EHR provided voicemail is full |
|  | Barriers for patients to arrange follow-ups | Relying on patients to arrange  follow-up by themselves |  |
| Solutions related to arranging a follow-up | Patient-Centered education | Provide information about the AHC |  |
|  | Standardize referral process | Provide electronic referral to the AHC |  |
|  |  | Referral to the Institute for Family Health if age is close to the limit for AHC referral |  |
|  | Correct documentation of contact information | Differentiate patients' personal phone number from parents’/guardians’ number in EHR | Full phone number information obtained during registration process |
|  | Interdisciplinary follow-up team | Send automatic notifications  of PED visit for STI to the AHC |  |
|  | Streamlined follow-up process at ED | Expedited follow-up at the PED itself | Walk-in center |
|  | Assistance for patients to arrange  an appointment |  |  |
|  | Standardize communication method | Correct documentation of contact information | Require contact number to be updated  when STI testing is ordered |
|  |  |  | Clarify patient's preferred contact method |
|  | Enable access to MyChart for all patients | Have residents assist with parents'/guardians’ and patients' access to MyChart |  |
|  |  | Provide MyChart setup assistance during triage |  |

**Table D. Pediatric emergency medicine fellows: challenges to and suggested solutions to overcoming the challenges in delivering STI care in the PED**

| **Challenge**  **or solution** | **Topics** | **Subtopics** | **Sub-subtopics** |
| --- | --- | --- | --- |
| **Medical history taking and documentation** | | | |
| Challenges related to medical history taking | Lack of privacy | Limited private space availability | No noise barrier in rooms |
|  |  |  | Curtained spaces |
|  |  |  | Difficulty ensuring privacy to obtain  truthful medical history |
|  |  |  | Risk of HIPAA violations |
|  |  | Separating companions (e.g. parents, guardian, partner) from patients | Lack of waiting space for companions (e.g. parents, guardian, partner) |
|  |  |  | Awkwardness feeling when separating companion from patient |
|  |  |  | Reluctance of the parent/guardian to  separate from patient |
|  | Communication challenges | Concern for STI not documented  by triaging nurse |  |
|  | Providers forgetting pertinent questions about sexual health |  |  |
| Solutions to medical history taking challenges | Enhance privacy | Ensure a proper space for obtaining  the medical history | Increase number of private rooms |
|  |  |  | Designation of waiting room  for the patient's companions |
|  |  |  | Utilize a space in the adult ED |
|  |  |  | Move patient to a less crowded space |
|  |  | Separate companions from patients |  |
|  |  | Use EHR privacy restrictions to  prevent parents/guardians from accessing certain sensitive notes |  |
| Challenges related to medical history documentation | Lack of privacy | Unclear EHR privacy restrictions of access to My Chart |  |
|  | Documentation challenges | Incomplete documentation | Documentation details included by physician depend on who will have access to the medical note (e.g., parent, guardian) |
| Solutions to Medical history documentation challenges | Enhance privacy | Clarify Epic Privacy Restrictions | Restricted access to MyChart  for parents/guardians |
|  |  |  | Improve security when logging into MyChart |
|  | Standardize the documentation process | Prepopulated template in EHR,  Smart phrases |  |
|  |  | Checklists | Accessible from mobile devices |
| **Conducting and documenting the physical examination** | | | |
| Challenges related to physical examination | Communication challenges | Room designation | Room designation changes are not always relayed to other staff |
|  | Delays in patient care | Unclear duty assignment | Difficulties with room cleaning |
|  |  | Inadequate assignment of duties | Physicians having to provide  patients with gown |
|  | Lack of privacy | Limited private space availability | Only one room equipped for  gynecology exams |
|  |  |  | The gynecology room occasionally  serves as an isolation area |
|  | Lack of functional equipment  and supplies availability | Gynecological bed not  properly functioning |  |
|  |  | Gynecological supplies are not  always available in the room |  |
|  |  | Difficulty performing a genitourinary exam in reclining chairs |  |
|  |  | Gynecological bed not  properly functioning |  |
| Solutions to physical examination challenges | Enhance privacy | Ensure a proper space | Utilize a space in the adult ED |
|  |  |  | Increase number of private rooms |
|  |  | Room designation changes | Limit the use of the gynecology room to patients with gynecological-related complaints only |
|  | Functional equipment  and supplies availability | Booster chairs for genitourinary exam |  |
|  |  | Detachable stirrups for stretchers |  |
|  |  | Ensure available gynecology  supplies in other rooms |  |
|  |  | Ensure gynecological bed  is properly functioning |  |
| Challenges related to physical examination documentation | Documentation challenges | Incomplete documentation | Prolonged time frame between patient  care and documentation |
|  |  |  | Difficulties remembering all details mentioned by patient |
|  |  |  | No specific template to  document physical findings |
|  |  |  | Undetailed description of physical findings |
|  |  | Heterogeneous documentation practices | Heterogeneous genitourinary examination description placement in chart |
| Solution to physical examination documentation challenges | Standardize the documentation process | Prepopulated template  in EHR, Smart phrases |  |
|  | Orientation and training for residents | Training on best  documentation practices |  |
| **STI testing** | | | |
| Challenges related to testing for STI | Extended wait for results | Patient psychological distress  while waiting for results |  |
|  |  | Time to results differs among the different HIV testing methods |  |
|  | Uncertainty regarding  appropriate test selection | Several different tests | Concerns about clean urine catch having a lower sensitivity for chlamydia and gonorrhea |
|  | Infrequent testing for trichomonas | No trichomonas test availability | Trichomonas test is not within the order set |
|  |  | Lack of knowledge  about trichomonas testing | Uncertainty regarding  appropriate test selection |
|  |  |  | Lack of knowledge about how to test |
|  |  |  | Unclear Trichomonas testing indication |
|  | Delays on labels printing | Challenges with label printing |  |
|  | Unclear sample handling process | Unclear sample labeling process | Delayed updates in processing status |
| Solutions to challenges related to testing for STI | Implementation of self-sample  collection is feasible | Offer self-swab as a routine procedure |  |
| **STI empiric treatment** | | | |
| Challenges related to STI treatment | Problems adhering to treatment | Access to medications | Long wait times to first medication  dose in PED from the hospital pharmacy |
|  |  |  | Pharmacy accessibility problems |
|  |  | Difficulty completing a  longer medication course |  |
|  | Lack of privacy | Confidentiality concerns | Insurance contacting parents/guardians |
|  |  |  | Incorrect contact information for patient |
|  | Duration of sexual abstinence  during the treatment |  |  |
|  | Unclear indications on who  to treat empirically |  |  |
|  | Discrepancies in regimen selection | Lack of awareness about  guidelines changes |  |
|  |  | Inter-provider variability | Need for selection of a third medication |
| Solutions to challenges related to STI treatment | Expedite patient's treatment | Provide full course of medications for free from the PED |  |
|  | Expedite partner treatment | Provide medications for extra  days to give to partners |  |
|  | Selection of patients to treat empirically | Shared decision for empirical treatment in patients with unclear STI diagnosis |  |
|  |  | Treat first if high STI suspicion |  |
|  | Provide education about  medication compliance | Provide education again during results call with physician assistant |  |
|  | Instructional documents for  treatment regimens | Make accessible through the Intranet | Make accessible guidelines and  protocols about pediatric EM |
| **Arrangement of follow-up visits** | | | |
| Challenges related to arranging a follow-up | Barriers for patients to arrange follow-ups | Relying on patients to arrange follow-ups by themselves |  |
|  |  | Low health literacy |  |
|  |  | Lack of assistance for patients to arrange an appointment |  |
|  |  | Language barriers |  |
|  | Barriers for physicians to assist in follow-up arrangements | Difficulty contacting patient's  primary care provider if  not Mount Sinai affiliated |  |
|  | Inability to keep track of patient  follow-up post-PED discharge | Not knowing about what happens after the patient leaves the PED |  |
| Solutions to challenges related to arranging a follow-up | Assistance for patients to  arrange an appointment while  patient in PED | Administrative staff to  schedule appointments |  |
|  |  | Appointment scheduler with visual signaling in the PED track board when appointment has been arranged |  |
|  | Systems for physicians to assist in follow-up arrangements | Special follow-up appointment slots for patients discharged from PED |  |
|  | Standardize referral process | Reminder before the appointment date | Automated calls to patients |
|  |  |  | Automated text messages to patients |
|  |  |  | Physician assistants to remind during  their results call with patients |

**Table E. Pediatric emergency department nurses: challenges to and suggested solutions to overcoming the challenges in delivering STI care in the PED**

| **Challenge or solution** | **Topics** | **Sub-topics** | **Sub-subtopics** |
| --- | --- | --- | --- |
| **Triage and initial assessment** | | | |
| Challenges related to triaging | Lack of privacy | Limited private space availability | No noise barrier in rooms |
|  |  |  | Crowding in the PED |
|  |  |  | The gynecology room occasionally serves  as an isolation area |
|  |  | Separating companions (e.g. parents, guardian, partner) from patients | Reluctance of the parent/guardian  to separate from patient |
|  |  | Confidentiality concerns | Parents’/guardians’ access to EHR |
|  | Communication challenges | Inconsistent sexual history responses  based on questioning method | Patients' limited knowledge about STI symptoms |
|  |  |  | Patients failing to disclose their symptoms |
|  |  |  | Male patients tend to be more reserved |
|  |  |  | Patients change their complaints after being triaged |
| Challenges related to identifying a patient | Communication challenges | Inconsistent sexual history responses  based on questioning method | Patients failing to disclose their symptoms |
|  |  |  | Adolescent unawareness of sexual health |
|  |  |  | Patients reporting vague symptoms |
|  |  |  | Patients' limited knowledge about STI symptoms |
|  | STI screening questions are not standardized |  |  |
| Solutions to challenges related to triage and identification of patient | Enhance privacy | Ensure a proper space | Designate private rooms for patients  with genitourinary complaints |
|  |  |  | Limit the use of the gynecology room to patients with gynecological-related complaints only |
|  |  |  | Front desk staff to move patient to private space when requesting to "speak to a doctor" |
|  |  | Use EHR privacy restrictions to prevent parents/guardians from accessing  certain sensitive notes |  |
|  | Enhanced triage protocol  for STI patients | Standardize STI screening during triage | Include STI questions in the  primary assessment section in the EHR |
|  | Communication enhancement | Implement code language to communicate sensitive complaints discreetly |  |
| **Preparation of patient for the history and physical examination** | | | |
| Challenges with patient preparation | Delays in patient care | Difficulties with chaperone availability | Nursing staff shortage |
|  |  |  | Unclear duty assignment |
|  | Lack of privacy | Limited private space availability | Only one room equipped for gynecology exams |
|  |  |  | Curtained spaces |
|  |  |  | No noise barrier in rooms |
|  |  |  | The gynecology room is used as a regular  patient room, not solely for examination |
|  | Adolescents may feel they don't belong in the PED | The room designation  does not consider age, which can place adolescents with STI complaints next to younger children within earshot |  |
|  | Difficulties with examination  room preparation | Nursing staff shortage |  |
|  |  | Physicians not preparing rooms  on their own |  |
|  | Reluctance of patients to be physically examined |  |  |
|  | Difficulties with patient preparation | Forgetting to ask the patient if it is  their first gynecology exam | Inadequate mental preparation for the  patient before a gynecological exam |
|  |  | Lack of standardized  patient preparation protocol | Inter-provider variability with examinations |
| Solutions to challenges with patient preparation | Change in workflow | Wait to place patients with genitourinary complaints in a private room instead of immediate placement in non-private area | Limit the use of the gynecology room to patients with gynecological-related complaints only |
|  |  |  | Use the room exclusively for physical  examinations, taking turns accordingly |
|  |  | Use urine pans as boosters during genitourinary examination |  |
|  |  | Separating companions (e.g. parents, guardian, partner) from patients | Ask sensitive questions only after the  companion leaves the room |
|  | Enhance privacy | Ensure a proper space for obtaining the history and physical examination | Use a privacy screen |
|  |  | Increase number of private rooms |  |
|  | Clarify roles | Chaperone duty distribution among staff |  |
|  | Orientation and training for nurses | Training on communication skills | Enhance the ability to  communicate about sensitive topics |
|  | Standardize testing process | Correct collection of urine samples |  |
| **Carrying out orders for STI testing** | | | |
| Challenges with carrying out orders for testing a patient | Unclear sample handling process | Unclear sample labeling process | Problems with unlabeled samples |
|  |  | Inadequate urine sample handling | Cancelled tests due to using the wrong tube |
|  | Sample collection challenges | Nurses are too busy to scan labels  before sending out sample | Nursing staff shortages |
|  |  | Delays in sample collection | Extended delay until the patient urinates |
|  |  | The type of required sample is  not displayed in EHR | Cancelled tests due to collecting the wrong sample |
|  | Test processing challenges | Some tests are not performed on a sample because the orders are not entered in EHR at the time of pickup |  |
| Solutions to challenges related to carrying out orders for testing | Orientation and training for nurses | More genitourinary and  gynecology training | Training about extragenital testing and  self-collected swabs |
|  | Functional equipment and supplies availability | Ensure label and wristbands availability |  |
|  | Workforce expansion | Hire more nurses |  |
|  | Save urine in a cup in case a  new test is needed |  |  |
|  | EHR improvements | Orders flagged showing the  test collection method needed |  |
| **Carrying out orders for STI treatment** | | | |
| Challenges carrying out orders for treating a patient | Challenges related to medication administration | Ceftriaxone injection is painful |  |
|  | Challenges related to  medication preparation | Confusion about ceftriaxone  dosing and mixing changes | Not formally informed about protocol changes |
|  |  | Unfamiliar with the specifics of mixing lidocaine and ceftriaxone |  |
|  | Delays in patient care | Delays caused by physicians  forgetting to order lidocaine |  |
| Solutions to challenges carrying out orders for treating a patient | Changes in medication preparation | Preparing ceftriaxone with lidocaine |  |
|  | Changes in medication administration | Explore intravenous administration as  an alternative to intramuscular |  |
|  | Orientation and training for nurses | Training on preparing ceftriaxone  with lidocaine | Clarify what type of lidocaine can  be mixed with ceftriaxone |

Key: STI (sexually transmitted infection), PED (pediatric emergency department), ED (emergency department), EHR (electronic health record), AHC (Adolescent Health Center)
